# Prevalence and Associated Risk Factors of Falls among Rural Community-Dwelling Older People: A Cross-Sectional Study from Southern Sri Lanka

**DOI:** 10.1155/2019/2370796

**Published:** 2019-05-28

**Authors:** Nirmala Gamage, Nirmala Rathnayake, Gayani Alwis

**Affiliations:** ^1^Department of Nursing, Faculty of Health Sciences, KAATSU International University, Sri Lanka; ^2^Department of Nursing, Faculty of Allied Health Sciences, University of Ruhuna, Sri Lanka; ^3^Department of Anatomy, Faculty of Medicine, University of Ruhuna, Sri Lanka

## Abstract

Falls may cause devastating consequences in older people. Conducting surveys on falls and factors associated with falls will inform better preventive health practices among older people to improve their quality of life. This study aimed to assess the prevalence and associated risk factors of falls and recurrent falls among rural community-dwelling older people in Southern Sri Lanka. A cross-sectional study was conducted in Nagoda Divisional Secretariat area, Galle, with 300 participants (females=175) aged 65 years and above. An interviewer-administered questionnaire was used to collect the data. To assess the prevalence of falls, participants were asked if they had fallen in the past year, and if so how many times. If any individual reported two or more falls, it was considered a recurrent fall. Biological, behavioral, environmental, and socioeconomic factors were documented as potential risk factors for falls. Multivariate logistic regression was performed with adjusted Odds Ratio (OR). Mean (SD) age was 73 (6.7) years. The prevalence of falls and recurrent falls were reported as 34.3% (95%CI; 29.03-40.04) (n=103) and 9.6% (95%CI; 6.68-13.73) (n=29), respectively. Out of 103 fallers, 37 (35.9%) had sustained injuries, with 40.5% affecting the wrist and 24.3% the hip. The associated factors for falls were age (OR=0.1), gender (OR=3.0), diabetes mellitus (OR=2.7), balance or gait problems (OR=4.2), use of antihypertensive medication (OR=0.2), and use of at least one long-term medication (OR=3.5). Associated factors for recurrent falls were age (OR=0.3), hypertension (OR=3.7), balance or gait problems (OR=3.8), sleep disturbances (OR=2.0), use of antihypertensive medication (OR=0.1), and use of at least one long-term medication (OR=3.4). A high prevalence of falls and low prevalence of recurrent falls among older people were observed. Biological and behavioral factors were reported as associated risk factors. This information is important to inform strategies to prevent falls in older people in Sri Lanka.

## 1. Introduction

With an increase in the elderly population, falls and fall-related injuries among older people have become a major public health concern in many countries. In Sri Lanka, the population over the age of 65 years is increasing and has been reported as 9.67% [1]. Increase of older people in a community creates a variety of public health issues, especially in developing countries, because of the difficulties in maintaining their health due to lack of resources.

A fall is defined as “in-advertently coming to rest on the ground, floor or other lower level, excluding intentional change in position to rest in furniture, wall or other objects” [2]. According to World Health Organization (WHO) statistics, every year 28-35% of people over 65 years and 32-42% of people over 70 years experience falls [2]. However, lower incidence of falls has been reported in developed countries such as UK, China, and Japan where rates have been reported as 22.4%, 19.3%, and 20% per year, respectively [2]. Falls in older people may cause fractures and soft tissue injuries leading to disability, loss of independence, and increased mortality [3]. In addition to falls related injuries, many older people experience emotional problems such as loss of confidence, fear, and anxiety that may further restrict their day-to-day activities [4]. Recurrent falls also have become a significant concern among older people. If an individual experienced two or more falls within a period of twelve months, it is considered as recurrent falls [5].

In Sri Lanka, 25.8% of community dwelling older people in Colombo had experienced falls during the preceding year [6]. Another hospital-based study of older people in Colombo reported that 23.3% of the people over 65 years experienced fall in a year [7]. Both studies further reported that high disability level and environmental risk factors were associated with falls [6]. However, both studies in Sri Lanka were carried out in the capital city of the country.

Risk factors of falls are multifactorial and have been categorized into four dimensions as biological, behavioral, environmental, and socioeconomic factors by the WHO [2]. The diverse nature of risk factors for falls indicates the importance of identification of fall associated risk factors among older people to prevent future falls and their consequences [8]. Studies focused on rural communities where the individual socioeconomic status, health status, and availability of health facilities are clearly different from the urban communities in Colombo are rare. With this background, the current study was planned to estimate the prevalence of falls and recurrent falls and secondly to identify associated risk factors for falls and recurrent falls among community dwelling older people living in a rural area of Southern Sri Lanka.

## 2. Materials and Methods

### 2.1. Study Design, Setting, and Sample Size

This cross-sectional household survey was conducted, during April and May 2015, with the participation of 300 community dwelling individuals aged 65 years and above from Nagoda Divisional Secretariat (DS) area, Galle, Sri Lanka. The cluster sampling method was used to achieve the required study sample from five out of 53 Grama Niladhari (GN) divisions that are under the administration of Nagoda DS office covering 53829 inhabitants with 5776 (10.73%) older people over 65 years of age [9].

The sample size calculation for the study was based on the prevalence of falls among community dwelling older people in Colombo, by using the falls prevalence of 25.8% in the previous year [6]. Precision was considered as 5% (±0.05). Considering a nonresponse rate of 10%, the calculated sample size was 294 and 300 individuals were recruited as final sample size. The individual cluster size was considered as 60 participants and the design effect was considered as “1” assuming all clusters are equal in nature [10]. The sociodemographic statuses of the individuals living in all the GN divisions of this specific DS area are almost equal [9].

Individuals without any acute illness and who could understand the questionnaire were eligible to participate in the study.

### 2.2. Data Collection and Measures

The data were collected at the participants' residence from the individuals who met the inclusion criteria with the support of a family member for the accuracy of the data. A convenient and adequate time was given to respond to the questions, without interfering their comfort and day-to-day activities. A pretested, interviewer administered questionnaire was administered by a single investigator while observing the existing facilities and falls risk at the home environment.

Sociodemographic profile evaluated the age, gender, marital status, educational status, and living companion. The prevalence of falls in the past year was assessed by asking “have you fallen in the last 12 months” and “if so how many times in the previous 12 months,” respectively. Participants were characterized as recurrent fallers if they had reported two or more falls in the previous 12 months. In the events of recurrent falls, time gap between first and second fall was also documented. Characteristics of falls were assessed on several factors including place, location of the fall at home and time of the fall, and whether they experienced any injury or fracture as a result of the fall, by asking “did you meet a doctor as a result of fall.” Risk factors for falls were assessed according to WHO defined biological, behavioral, environmental, and socioeconomic factors [2]. Biological factors included age, gender, and chronic medical conditions such as hypertension, diabetes mellitus, cardiac diseases, hyperlipidemia, epilepsy, chronic pulmonary disease, arthritis, and vertigo. These were assessed by asking if they had been diagnosed by a physician. Self-stated vision impairment, sleep disturbances and balance, gait, or foot abnormalities were documented. Behavioral factors included intake of long-term medications including antihypertensive, hypoglycemic, and cardiac/lipid lowering drugs or any other medications, nutritional status, average time of sun exposure per day, usage of walking aids, consumption of alcohol, and active smoking status.

Comorbidities and long-term medication related data were collected from medical reports. Diagnosis cards, doctors' notes, clinic records, and self-explanations of participants were also accepted when the medical records were not available. Such cases were cross-checked with a family member at home. Only the diagnosis was recorded and, for medication, the type and number of medications consumed were recorded.

Nutritional status was evaluated using a food frequency questionnaire by creating scores for self-reported consumption of six selected commonly available and used food items (meat/fish/eggs, vegetables, milk and dairy products, green leaves, fruits, and cereals) per week. The maximum points for a food item were three with a minimum of zero. Total maximum points which could be obtained were 18 for the six food items. Individuals who scored 0-9 points were considered to have unsatisfactory nutritional status and those who scored 10-18 points were considered to have satisfactory nutritional status.

Environmental factors that increase the falls risk such as light source at home, slippery surfaces, and water source inside and outside the home were also evaluated. Finally, socioeconomic risk factors such as low income and low education were also recorded. In addition, individuals were asked about their living companion (alone versus living with others) at home.

### 2.3. Statistical Analyses

Descriptive analyses were performed for demographic characteristics, falls related characteristics and evaluated falls associated factors and presented as mean (SD) or frequency (%). The prevalence of falls and recurrent falls were calculated as frequency (%) and presented with 95% CI. Prevalence rates by the various evaluated risk factors were evaluated with Chi square test of independence or Fisher's exact test, to assess the associations of falls and recurrent falls with different fall associated risk factors. Univariate logistic regression analysis was performed with unadjusted (crude) OR at 95% CI to identify which variables were considered as associated factors for falls and recurrent falls. Multiple logistic regression (backward-conditional) was applied for the variables that were significant in univariate analysis for retaining only the most significant associated risk factors for falls and recurrent falls and presented with adjusted OR and 95% CI. In the logistic regression analysis, age was defined as young older people and old older people if the participant's age was between 65-74 years and ≥ 75 years, respectively. Statistical analyses were performed using Statistical Package of Social Sciences (SPSS) version 20.0. P value < 0.05 was regarded as acceptable.

### 2.4. Ethical Considerations

The ethical approval for the study was obtained from the Ethical Review Committee of Faculty of Medicine, University of Ruhuna, Sri Lanka. Written informed consent was obtained from each study participant before the administration of questionnaire.

## 3. Results

### 3.1. Sociodemographic Characteristics of the Study Sample

The study sample consisted of 300 participants aged 65 years and above (range 65-99) with a mean (SD) age of 73 (6.7) years. Of them, 175 (58.3%) were females. Majority (n=268, 89.3%) were living with their spouse and/or children (Table 1). There was no significant difference of socioeconomic status between fallers and nonfallers or recurrent fallers and nonrecurrent fallers except the education level.

### 3.2. Medical Conditions and Related Factors among the Study Sample

Commonly reported diagnosed medical conditions were hypertension (n=124, 41.3%), diabetes mellitus (n=57, 19.0%), cardiac disease (n=34, 11.3%), arthritis (n=24, 8.0%), and hyperlipidemia (n=22, 7.3%). Commonly reported perceived health problems affecting day-to-day activities were vision problems (n=230, 76.7%), balance or gait problems or foot abnormalities (n=109, 36.3%), and sleep problems (n=144, 48.0%). Commonly reported long-term medications were antihypertensive (n=109, 36.3%), hypoglycemic drugs (n=49, 16.3%), and lipid lowering drugs (n=45, 15.0%).

### 3.3. Prevalence of Falls, Recurrent Falls, and Characteristics of Falls

The prevalence of falls within the last 12 months was 34.3% (95% CI; 29.03-40.04) (n=103). Among the fallers, 71 (68.9%) were females and 60 (58.3%) were aged between 65 and 74 years. Out of 103 (34.3%) falls during the previous year, 74 (71.8%) had experienced at least one fall, 10 (9.7%) had two, and 19 (18.4%) had three or more falls (Table 2). The prevalence of recurrent falls was 9.67% (95% CI; 6.68-13.73) (n=29). Among them 18 (62.1%) reported the time gap between the first and second fall as less than 6 months (Table 2). Out of 29 (9.67%) recurrent falls, 20 (68.9%) were in females and 7 (24.1%) were in people aged 75 years and above.

The majority of falls occurred in the home environment. Nearly half of the falls occurred during the daytime. Among the injuries following falls, sustained soft tissue injuries including bruises, abrasions, and swelling at wrist and at hip were common (Table 2).

Comparisons of various evaluated fall related factors between fallers and nonfallers are shown in Table 3. Most of the biological and behavioral factors were significantly associated with falls prevalence (p<0.05). Comorbid diseases were significantly higher among fallers, with having at least one chronic medical condition, vision problems affecting their day-to-day life, balance problem or foot abnormality, and sleep problems. Usage of medication was higher among fallers using at least one long term medication. Among the environmental factors, floor material, light source, and water source at home were also significantly associated with falls (p<0.05). Except education level, other evaluated socioeconomic factors were not associated with falls as evaluated by Chi square test or Fisher's exact test (Table 3).

Comparisons of various evaluated fall related factors between recurrent and nonrecurrent fallers are shown in Table 4. Most of the biological and behavioral factors were significantly associated with recurrent falls as evaluated by Chi square test or Fisher's exact test (p<0.05). Vision impairment, gait problems, sleep problems, and usage of long-term medications were significantly higher among the recurrent fallers than the nonrecurrent fallers (p<0.05). None of the environmental factors was significantly associated with recurrent falls and only the low education was associated out of the socioeconomic factors (Table 4).

### 3.4. Univariate Logistic Regression Analysis of Associated Factors for Falls and Recurrent Falls

Table 5 shows the associated risk factors with crude OR for falls prevalence.  Table 6 shows the associated risk factors with crude OR for recurrent falls prevalence.

### 3.5. Multivariate Logistic Regression Analysis of Associated Risk Factors for Falls and Recurrent Falls

All significant factors that were identified with univariate logistic regression analyses were entered into a multivariate logistic regression model to identify the independent associated risk factors for falls and recurrent falls (Tables 5 and 6).

The significant risk factors for falls were age (OR=0.1, 95% CI; 0.0-0.3), gender (OR=3.0, 95% CI; 1.5-5.9), diabetes mellitus (OR=2.7, 95% CI; 1.2-6.3), balance or gait problems (OR=4.2, 95% CI; 2.0-8.4), using antihypertensive medication (OR=0.2, 95% CI; 0.0-0.8), and use of at least one long-term medication (OR=3.5, 95% CI; 1.2-10.4).

The significant risk factors for recurrent falls were age (OR=0.3, 95% CI; 0.1-0.6), hypertension (OR=3.7, 95% CI; 1.0-13.0), balance or gait problems (OR=3.8, 95% CI; 2.1-7.1), sleep disturbances (OR=2.0, 95% CI; 1.1-3.8), using antihypertensive medication (OR=0.1, 95% CI; 0.0-0.5), and use of at least one long term medication (OR=3.4, 95% CI; 1.2-9.7).

## 4. Discussion

The current study assessed the prevalence and associated risk factors for falls and recurrent falls among rural community-dwelling older people in Galle, Sri Lanka. The prevalence of falls in the present study was 34.3% which is a comparatively high prevalence compared to the studies reported from urban community in Sri Lanka [6, 7], in developed countries such as Canada, UK, China, Japan, and Australia [3, 4, 11–13], and in a developing country like India [14]. The prevalence of recurrent falls was 9.67%, which is much lower than the previously reported values from Sri Lanka [6], USA [15], and Ecuador [16] and higher compared to China [13]. Recall bias in the retrospective studies, especially in older people, is one possible explanation for the large discrepancy. However, the prevalence of falls among older people in the current study lies within the range of 28%-35% reported by the WHO Global Report on Falls Prevention [2]. One possible reason for higher falls prevalence in the current study compared to the urban community dwellers in Sri Lanka [6, 7] would be rural community dwellers having to perform many tasks during their day-to-day life including farming and manual labor, which may increase the risk of falling.

Higher prevalence of falls among females compared to the males is consistent with many studies [6, 14, 17]. Literature suggests that fall reporting accuracy is low, because some individuals, especially men, perceive the fall as stigmatized event and are reluctant to report them accurately [18]. However, we obtained supplementary data from a family member who was present at the time of data collection to minimize errors of gathered data.

One possible reason for the higher prevalence of falls among young older people compared to the old older people may be young older people are more active, self-confident, and engaging in more risky activities which increase the tendency to fall. Higher prevalence of falls has been reported during daytime at the home environment. This may be because older people spend most of their time at home and are active during daytime.

The percentage of individuals who sustained fall related injury was higher in our study than reported from Ecuador [16] and India [19] and lower than in China [20]. Reasons for these discrepancies among different populations could be multifactorial and beyond the scope of this study.

Out of the associated risk factors evaluated, age, gender, presence of diabetes mellitus, presence of balance or gait problems or foot abnormalities, and use of antihypertensive and at least one long-term medication remained as associated risk factors for falls. Among the fallers, the recurrent fallers are more prone to have hypertension, sleep problems, balance and gait abnormalities, and antihypertensive medication. The study highlighted the importance of hypertension, balance or gait problems, sleep problems, and use of antihypertensive medication for increased risk of recurrent falls. Prior study also reported that hypertensive patients presented with recurrent falls [15]. In addition, in the majority of recurrent fallers, the gap between first and second fall was less than 6 months. This indicates the severity of the issue.

Exposure to side effects and drug compliance for these medications may be another reason for the increased risk of falls in young older group as diseases are first experienced during young adult age. The finding that falls risk increases with age is consistent with the literature [14, 17]. It is also noted that balance or gait problems and foot abnormalities were significant in fallers and recurrent fallers, which is consistent with other studies [14, 15, 17].

Some of the identified associated risk factors for falls in the current study are modifiable. Early identification of predisposing factors and referral to relevant care centers may reduce risk of falls. Further, the older people who have balance or gait problems or foot abnormalities should be given more attention with a close observation. However, in the current study, none of the environmental or socioeconomic factors were associated with the prevalence of falls in logistic regression analysis. Our finding is inconsistent with the cited studies conducted in Colombo in which they have reported that up to 40% of fall risk can be reduced by eliminating risk factors at home environment [7]. Therefore, the impacts of environmental risk factors and socioeconomic factors on fall prevalence need to be evaluated in future prospective studies.

Our study has major strengths. This is the first ever study done in Sri Lanka which elaborates the prevalence of falls, prevalence of recurrent falls, and associated risk factors in community dwelling older people living in a rural area. Further, we were able to identify the variety of risk factors while observing the home environment during data collection. Even though self-stated vision impairment was not retained as a risk factor for falls and recurrent falls in the current study, many individuals were reported with self-stated vision impairment affecting day-to-day activities. We were able to refer them for relevant medical care for the proper assessment of vision status. However, there are also several limitations of this study. First, the injury prevalence and disability levels following falls were reported by the self-stated answers and most of the older people did not have diagnosis cards or X-ray reports to assess the exact fragility fracture rate following falls. Second, we could not study the side effects of individual medications and drug compliance. Finally, this investigation is a cross-sectional study. The cross-sectional design could not determine the causal relationship between falls and associated risk factors. Therefore, future prospective investigations with proper assessment of existing physical activity levels, physical performances, muscle strength, balance and gait speed, and vision and cognitive impairments are needed to identify the determinants of falls and recurrent falls of older people.

## 5. Conclusions

A high prevalence of falls and low prevalence of recurrent falls among rural community-dwelling older people were evident in this cross-sectional study. Most of the associated risk factors for falls and recurrent falls can be modified. This study emphasizes the importance of early detection of health problems and behavioral characteristics that can increase the risk of falls as the falls can have a major impact on quality of life of older people.

## Figures and Tables

**Table 1:** Demographic characteristics of the study sample (n=300).

Demographic characteristics		Number (%)
Age group	65-74 years	200 (66.7)
	75 years and above	100 (33.3)

Gender	Male	125 (41.7)
	Female	175 (58.3)

Level of education	No schooling	23 ( 7.7)
	Primary education	93 (31.0)
	Secondary education	71 (23.7)
	Ordinary Level	77 (25.7)
	Advanced Level	31 (10.3)
	Diploma/Degree	05 (1.7)

Marital status	Married	201 (67.0)
	Single	19 ( 6.3)
	Widowed	73 (24.3)
	Divorced	04 (1.3)
	Separated	03 (1.0)

Living companion	With spouse or children	268 (89.3)
	Alone	32 (10.7)

Monthly income (Sri Lankan Rupees)	< 10000	132 (44.0)
	10000-20000	149 (49.7)
	20000-50000	19 (6.3)

**Table 2:** Falls related information (n=103).

Details about falls		Frequency (Percentage %)
Number of falls within last 12-month period	One	74 (71.8)
	Two	10 (9.7)
	Three or more	19 (18.4)

*∗*Time gap between first and second fall (n= 29)	<6 months	18 (62.1)
	6-12 months	11 (37.9)

Time of last fall	Early morning	25 (24.3)
	Day time	54 (52.4)
	Night	24 (23.3)

Place of fall	At home environment	74 (71.8)
	Outside home environment	29 (28.2)

Location of fall at home	When stepping stairs	35 (34.0)
	On the floor	17 (16.5)
	In the garden	17 (16.5)
	Toilet	14 (13.6)
	Bathing place	12 (11.7)
	When getting out of bed	7 (6.8)
	Chair	1 (1.0)

Experienced fall related injuries	Yes	37 (35.9)
	No	66 (64.1)

*∗∗*Injury site (n= 37)	Wrist	15 (40.5)
	Hip	9 (24.3)
	Ankle	5 (13.5)
	Skull	3 ( 8.1)
	Knee	2 ( 5.4)
	Femur shaft	2 ( 5.4)
	Backbone	1 ( 2.7)

**Table 3:** Comparison of risk factors between fallers and nonfallers (n=300).

Characteristic		Fallers n=103	Nonfallers n=197	Significance (p value)
*Biological factors*				

Age	65-74 years	60 (58.3)	40 (20.3)	< 0.001
	75 years and above	43 (41.7)	157 (79.7)	

Gender	Male	32 (31.1)	93 (47.2)	0.007
	Female	71 (68.9)	104 (52.8)	

Presence of at least one chronic medical condition		81 (78.6)	102 (51.8)	< 0.001

Presence of more than one chronic medical condition		42 (40.8)	44 (22.3)	0.001

Presence of more than two chronic medical conditions		14 (13.6)	9 (4.6)	0.005

Diabetes Mellitus		31 (30.1)	26 (13.2)	< 0.001

Cardiac diseases		20 (19.4)	14 (7.1)	0.001

Hypertension		56 (54.4)	68 (34.5)	0.001

Hyperlipidemia		7 ( 6.8)	15 (7.6)	0.79

Vertigo/Dizziness		7 ( 6.8)	4 (2.0)	0.04

Arthritis		9 ( 8.7)	15 (7.6)	0.73

Chronic pulmonary diseases		7 ( 6.8)	10 (5.1)	0.54

Epilepsy		1 ( 1.0)	5 (2.5)	0.35

Vision impairment affecting day-to-day activities		93 (90.3)	137 (69.5)	< 0.001

Balance/gait problem or foot abnormality		68 (66.0)	41 (20.8)	< 0.001

Problem with sleep such as insomnia		72 (69.9)	72 (36.5)	< 0.001

*Behavioral factors*				

Usage of at least one long term medication		64 (62.1)	75 (38.1)	< 0.001

Usage of more than one long term medication		32 (31.1)	30 (15.2)	0.001

Usage of more than two long term medications		8 (7.8)	9 ( 4.6)	0.25

Antihypertensive drugs		46 (44.7)	63 (32.0)	0.03

Oral hypoglycemic drugs		28 (27.2)	21 (10.7)	< 0.001

Cardiac/lipid lowering drugs		23 (22.3)	22 (11.2)	0.01

Steroids		8 ( 7.8)	8 ( 4.1)	0.17

Consumption of alcohol		18 (17.5)	53 (26.9)	0.06

Smoking		11 (10.7)	26 (13.2)	0.52

Time duration of exposure to sunlight per day	≤ 3 hours	74 (71.8)	84 (42.6)	< 0.001
	>3 hours	29 (28.2)	113 (57.4)	

Nutritional status	Satisfactory	47 (45.6)	59 (29.9)	0.007
	Unsatisfactory	56 (54.4)	138 (70.1)	

Usage of walking aids		25 (24.3)	6 ( 3.0)	< 0.001

Use of spectacles		53 (51.5)	91 (46.2)	0.38

*Environmental factors*				

Floor material	Cement/tile	92 (89.3)	195 (99.0)	< 0.001
	Other	11 (10.7)	2 (1.0)	

Light source of the house	Electricity	95 (92.2)	195 (99.0)	0.002
	Other	8 (7.8)	2 (1.0)	

Water source	Inside home	78 (75.7)	178 (90.4)	0.001
	Outside home	25 (24.3)	19 (9.6)	

*Socioeconomic factors*				

Level of education	Grade 5 or below	51 (49.5)	65 (33.0)	0.005
	Beyond grade 5	52 (50.5)	132(67.0)	

Living arrangement	With spouse or children	90 (87.4)	178 (90.4)	0.42
	Alone	13 (12.6)	19 (9.6)	

Monthly income (Sri Lankan Rupees)	< 20000	100(97.1)	181 (91.9)	0.07
	20000 or more	3 (2.9)	16 (8.1)	

**Table 4:** Comparison of risk factors between recurrent fallers and nonrecurrent fallers (n=300).

Characteristic		Recurrent fallers n=29	Nonrecurrent fallers n=271	Significance (p-value)
*Biological factors*				

Age	65-74 years	22 (75.9)	78 (28.8)	< 0.001
	75 years and above	7 (24.1)	193 (71.2)	

Gender	Male	9 (31.0)	116 (42.8)	0.22
	Female	20 (69.0)	155 (57.2)	

Presence of at least one chronic medical condition		27 (93.1)	156 (57.6)	< 0.001

Presence of more than one chronic medical condition		18 (62.1)	68 (25.1)	< 0.001

Presence of more than two chronic medical conditions		9 (31.0)	14 (5.2)	< 0.001

Diabetes Mellitus		13 (44.8)	44 (16.2)	< 0.001

Cardiac diseases		9 (31.0)	25 (9.2)	< 0.001

Hypertension		21 (72.4)	103 (38.0)	< 0.001

Hyperlipidemia		1 (3.4)	21 (7.7)	0.39

Vertigo/Dizziness		4 (13.8)	7 (2.6)	0.002

Arthritis		2 (6.9)	22 (8.1)	0.81

Chronic pulmonary disease		4 (13.8)	13 (4.8)	0.04

Epilepsy		1 (3.4)	5 (1.8)	0.55

Vision impairment affecting day-to-day activities		27 (93.1)	203(74.9)	0.02

Balance/gait problems or foot abnormality		26 (89.7)	83 (30.6)	< 0.001

Problem with sleep such as insomnia		22 (75.9)	122 (45.0)	0.002

*Behavioral factors*				

Usage of at least one long term medication		22 (75.9)	117 (43.2)	0.001

Usage of more than one long term medication		14 (48.3)	48 (17.7)	< 0.001

Usage of more than two long term medication		6 (20.7)	11 (4.1)	< 0.001

Antihypertensive drugs		18(62.1)	91 (33.6)	0.002

Oral hypoglycemic drugs		12 (41.4)	37 (13.7)	< 0.001

Cardiac/lipid lowering drugs		10 (34.5)	35 (12.9)	0.002

Steroids		3 (10.3)	13 (4.8)	0.20

Consumption of alcohol		3 (10.3)	68 (25.1)	0.07

Smoking		2 (6.9)	35 (12.9)	0.34

Time duration of exposure to sunlight per day	≤ 3 hours	27 (93.1)	131 (48.3)	< 0.001
	>3 hours	2 (6.9)	140 (51.7)	

Nutritional status	Satisfactory	13 (44.8)	93 (34.3)	0.26
	Unsatisfactory	16 (55.2)	178 (65.7)	

Usage of walking aids		15 (51.7)	16 (5.9)	< 0.001

Use of spectacles		15 (51.7)	129 (47.6)	0.67

*Environmental factors*				

Floor material	Cement/tile	28 (96.6)	259 (95.6)	0.80
	Other	1 (3.4)	12 (4.4)	

Light source of the house	Electricity	28 (96.6)	262 (96.7)	0.97
	Other	1 (3.4)	9 (3.3)	

Water source	Inside home	22 (75.9)	234 (86.3)	0.12
	Outside home	7 (24.1)	37 (13.7)	

*Socioeconomic factors*				

Level of education	Grade 5 or below	17 (58.6)	99 (36.5)	0.02
	Beyond grade 5	12 (41.4)	172 (63.5)	

Living arrangement	With spouse or children	27 (93.1)	241 (88.9)	0.48
	Alone	2 ( 6.9)	30 (11.1)	

Monthly income (Sri Lankan Rupees)	< 20000	28 (96.6)	253 (93.4)	0.50
	20000 or more	1 ( 3.4)	18 (6.6)	

**Table 5:** Logistic regression analysis of risk factors for falls (n=300).

Risk factor	Univariate analysis			Multivariate analysis		
	Crude OR	95% CI OR	P value	Adjusted OR	95% CI OR	P value
*Biological factors*						

Age	0.1	0.1-0.3	<0.001	0.1	0.0-0.3	<0.001

Gender	1.9	1.2-3.2	0.008	3.0	1.5- 5.9	0.002

Presence of at least one chronic medical condition	3.4	1.9-5.9	<0.001	-	-	-

Presence of more than one chronic medical condition	2.3	1.4-4.0	0.001	-	-	-

Presence of more than two chronic medical conditions	3.2	1.3-7.8	0.001	-	-	-

Diabetes mellitus	2.8	1.5-5.1	0.001	2.7	1.2- 6.3	0.01

Ischemic heart diseases	3.1	1.5-6.5	0.002	-	-	-

Hypertension	2.2	1.3-3.6	0.001	-	-	-

Vertigo	3.5	1.0-12.3	0.04	-	-	-

Self-reported vision problem affecting day-to-day life	4.0	1.9-8.3	<0.001	-	-	-

Self-reported balance/gait problems or foot abnormality	7.3	4.3-12.6	<0.001	4.2	2.0- 8.4	< 0.001

Self-reported sleeping problems	0.2	0.1-0.4	<0.001	-	-	-

*Behavioral factors*						

Usage of at least one long term medication	2.6	1.6-4.3	<0.001	3.5	1.2- 10.4	0.02

Usage of more than one long term medication	2.5	1.4-4.4	0.002	-	-	-

Antihypertensive	1.7	1.0-2.8	0.03	0.2	0.0- 0.8	0.02

Hypoglycemic drugs	3.1	1.6-5.8	<0.001	-	-	-

Cardiac/Lipid lowering drugs	2.2	1.2-4.3	0.01	-	-	-

Exposure to sun	0.2	0.1-0.4	<0.001	-	-	-

Nutritional status	0.5	0.3-0.8	0.007	-	-	-

Usage of walking aids	0.1	0.0-0.2	<0.001	-	-	-

*Environmental factors*						

Floor material	11.6	2.5-53.6	0.002	-	-	-

Light source	8.2	1.7-39.4	0.009	-	-	-

Water source	3.0	1.5-5.7	0.001	-	-	-

*Socioeconomic factors*						

Level of education	0.5	0.3-0.8	0.006			

OR=Odds Ratio; CI=Confidence Interval.

The variables which are significant in the chi square or fisher's exact test with falls prevalence were used for univariate logistics regression analyses and were presented in this table.

The variables which were significant with the falls prevalence in univariate logistics regression were further evaluated with multivariate logistic regression and only the significant variables were presented in the table.

**Table 6:** Logistic regression analysis of risk factors for recurrent falls (n=300).

Risk factor	Univariate analysis			Multivariate analysis		
	Crude OR	95% CI OR	P value	Adjusted OR	95% CI OR	P value
*Biological factors*						

Age	0.1	0.1-0.3	<0.001	0.3	0.1- 0.6	0.001

Presences of at least one chronic medical condition	3.4	0.1-5.9	<0.001	-	-	-

Presence of more than one chronic medical conditions	2.3	1.4-4.0	0.001	-	-	-

Presence of more than two chronic medical conditions	3.2	1.3-7.8	0.008	-	-	-

Diabetes mellitus	2.8	1.5-5.1	0.001	-	-	-

Ischemic heart diseases	3.1	1.5-6.5	0.002	-	-	-

Hypertension	2.2	1.3-3.6	0.001	3.7	1.0- 13.0	0.04

Vertigo	3.5	1.0-12.3	0.04	-	-	-

Self-reported vision problem affecting day-to-day life	4.0	1.9-8.3	<0.001	-	-	-

Self-reported balance/gait problems or foot abnormality	7.3	4.3-12.6	<0.001	3.8	2.1- 7.1	<0.001

Self-reported sleeping problems	4.0	2.4-6.7	<0.001	2.0	1.1- 3.8	0.02

*Behavioral factors*						

Usage of at least one long term medications	2.6	1.6-4.3	<0.001	3.4	1.2- 9.7	0.01

Usage of more than one long term medications	2.5	1.4-4.4	0.002	-	-	-

Antihypertensive	1.7	1.0-2.8	0.03	0.1	0.0- 0.5	0.007

Hypoglycemic drugs	3.1	1.6-5.8	<0.001	-	-	-

Cardiac/Lipid lowering drugs	2.2	1.2-4.3	0.01	-	-	-

Exposure to sun	0.2	0.1-0.4	<0.001	-	-	-

Usage of walking aids	10.2	4.0-25.8	<0.001	-	-	-

*Socio-economic factors*						

Level of education	0.4	0.1-0.8	0.02	-	-	-

OR=Odds Ratio, CI=Confidence Interval

The variables which are significant in the chi square or fisher's exact test with recurrent falls prevalence were used for univariate logistics regression analyses and were presented in this table.

The variables which were significant with the recurrent falls prevalence in univariate logistics regression were further evaluated with multivariate logistic regression and only the significant variables were presented in the table.

## Data Availability

The data used to support the findings of this study are available from the corresponding author upon request.
